# Editorial: Function of vestibular and auditory systems in neurogenerative disorders

**DOI:** 10.3389/fneur.2025.1623371

**Published:** 2025-05-23

**Authors:** Tatyana Yakusheva, Aasef G. Shaikh

**Affiliations:** ^1^Department of Otolaryngology, Washington University School of Medicine, St. Louis, MO, United States; ^2^Department of Neurology, University Hospitals, Case Western Reserve University, Cleveland, OH, United States; ^3^Department of Biomedical Engineering, Case Western Reserve University, Cleveland, OH, United States; ^4^Functional Electrical Stimulation Center, Cleveland VA Medical Center, Cleveland, OH, United States

**Keywords:** vestibular function, neurodegeneration, balance, falls, imbalance

Dizziness, often precipitating imbalance and falls, remains a prevalent and consequential health issue worldwide. Its implications are particularly profound among aging populations, such as the burgeoning number of baby boomers. The burden of dizziness is not merely a matter of discomfort but a significant contributor to morbidity and, in some cases, mortality.

Central to many dizziness cases is dysfunction of the peripheral inner ear—the vestibular organ that plays a vital role in sensing and maintaining balance. The peripheral vestibular system comprises fluid-filled structures intricately involved in detecting angular velocity, head orientation, and linear acceleration through complex fluid dynamics. These processes are tightly regulated; disturbances, such as in Ménière's disease or endolymphatic hydrops, disrupt inner ear pressure and compromise function.

Signals from the peripheral vestibular apparatus are transmitted via reflex pathways—including the vestibular-ocular reflex—to the brain. Higher brain centers, involving the cerebellum, basal ganglia, and cerebral cortex, integrate this information to facilitate spatial navigation and posture control. Disruption at any level can result in dizziness, vertigo, and imbalance, highlighting the importance of understanding both peripheral and central contributions to vestibular function.

Recent research sheds light on various facets of vestibular pathology and its wider implications. For instance, studies comparing posterior canal cupulolithiasis and canalolithiasis reveal that both conditions impair vestibular function, evidenced by abnormal vHIT and VEMP results, without side-specific correlation—suggesting widespread peripheral involvement rather than localized damage.

Diagnostic advancements such as the cochlear hydrops analysis masking procedure (CHAMP) offer promising tools for detecting endolymphatic hydrops characteristic of Ménière's disease. Although variability persists in its diagnostic accuracy, CHAMP enhances our ability to study hydropic ear diseases and improve detection strategies.

Genetic investigations, including Mendelian randomization studies, have explored potential links between migraine and Ménière's disease. Findings indicate that migraine susceptibility does not causally increase the risk of MD, underscoring the complexity of vestibular disorders and their etiologies.

Surgical interventions, such as cochlear implantation, are evolving to preserve vestibular function. New electrode designs aim to minimize trauma; however, emerging evidence suggests that prolonged drug delivery from implant devices could induce vestibular hydrops, emphasizing the need for ongoing refinement to balance auditory and vestibular preservation.

Beyond peripheral mechanisms, the central vestibular system's role in disease states like Parkinson's disease (PD) is increasingly recognized. Vestibular dysfunction contributes to postural instability and falls in PD patients. Therapeutic strategies targeting vestibular rehabilitation have shown promise in alleviating these symptoms, potentially improving quality of life.

Moreover, innovative approaches such as rhythmic auditory cues (RAC) demonstrate therapeutic potential in modulating gait disturbances in PD. Studies reveal RAC can reduce gait asymmetry during steady walking, although effects vary with task complexity, suggesting personalized rehabilitative strategies are warranted.

In conclusion, understanding dizziness and vestibular pathology requires a comprehensive view that encompasses peripheral, central, and systemic factors. Advances in diagnostic and therapeutic modalities hold promise for reducing the impact of vestibular disorders across all ages, particularly as the population continues to age. Continued research is essential to unravel the complex interplay within the vestibular system and develop targeted interventions to improve patient outcomes.

